# Validity of a self-report survey tool measuring the nutrition and physical activity environment of primary schools

**DOI:** 10.1186/1479-5868-10-75

**Published:** 2013-06-13

**Authors:** Nicole Nathan, Luke Wolfenden, Philip J Morgan, Andrew C Bell, Daniel Barker, John Wiggers

**Affiliations:** 1 Hunter New England Population Health, Hunter New England Area Health Service, Newcastle; Locked Bag No. 10, Wallsend, NSW, 2287, Australia; 2 School of Medicine and Public Health, The University of Newcastle, Newcastle, NSW, 2308, Australia; 3 Priority Research Centre for Health Behaviour, The University of Newcastle, Newcastle, NSW, 2308, Australia; 4 Hunter Medical Research Institute, Newcastle, NSW, 2300, Australia; 5 Priority Research Centre in Physical Activity & Nutrition, Faculty of Education & Arts, The University of Newcastle, Newcastle, NSW, 2308, Australia

## Abstract

**Background:**

Valid tools measuring characteristics of the school environment associated with the physical activity and dietary behaviours of children are needed to accurately evaluate the impact of initiatives to improve school environments. The aim of this study was to assess the validity of Principal self-report of primary school healthy eating and physical activity environments.

**Methods:**

Primary school Principals (n = 42) in New South Wales, Australia were invited to complete a telephone survey of the school environment; the School Environment Assessment Tool – SEAT. Equivalent observational data were collected by pre-service teachers located within the school. The SEAT, involved 65 items that assessed food availability via canteens, vending machines and fundraisers and the presence of physical activity facilities, equipment and organised physical activities. Kappa statistics were used to assess agreement between the two measures.

**Results:**

Almost 70% of the survey demonstrated moderate to almost perfect agreement. Substantial agreement was found for 10 of 13 items assessing foods sold for fundraising, 3 of 6 items assessing physical activity facilities of the school, and both items assessing organised physical activities that occurred at recess and lunch and school sport. Limited agreement was found for items assessing foods sold through canteens and access to small screen recreation.

**Conclusions:**

The SEAT provides researchers and policy makers with a valid tool for assessing aspects of the school food and physical activity environment.

## Introduction

Schools have been recognised as an important setting for promoting child healthy eating and physical activity [1]. Ecological frameworks and empirical research suggest that a number of school characteristics such as their economic, policy and socio-cultural environment can impact upon these behaviours [2]. Moreover, the physical environment of schools can significantly impact upon the nutrition and physical activity behaviours of children [3-6]. For example, the availability of high kilojoule, low-nutrient dense foods such as soft-drinks and confectionary through school canteens, vending machines or fundraising activities have been associated with children consuming a higher intake of kilojoules and saturated fat and a lower intake of vegetables and fruit [1,4,5,7,8]. Similarly, the availability of open spaces (e.g. courts or fields) [9]; playground markings [10]; fixed outdoor equipment such as climbing structures [10,11], the availability of sports equipment [12] and provision of organized games or sports during recess and lunch [13] have been associated with higher levels of child physical activity at school.

Given the suggested influence of the school environment on children’s diet and physical activity, encouraging schools to create environments supportive of such behaviours has been a focus of Government obesity prevention policies and programmes in a number of countries [14]. For example, some jurisdictions in The United States and Canada have prohibited the sale of foods which are high in sugar and saturated fats through vending machines and school meals and have mandated the teaching of physical education and the amount of class time dedicated to moderate-vigorous activities [1]. Similarly, a number of Australian States and Territories have released mandatory Government policies banning the regular sale of unhealthy foods and beverages from canteens, school events and fundraisers [15]. Furthermore, they have stipulated the minimum number of hours children must participate in physical education and sport [16]. Specifically within New South Wales all Government schools are restricted from selling some sugar sweetened drinks and foods that are high in saturated fats and or sugar and or salt such as confectionary, fried foods and sweet and savoury snack foods [17]. In addition all Government primary schools are required to allocate 120 minutes of planned physical activity each week, inclusive of school sport, in Years 3 to 6 [16].

Valid tools to measure school environment characteristics are needed to accurately assess the nature and prevalence of nutrition and physical activity promoting characteristics of schools, the trends and changes over time in the prevalence of such characteristics, and to evaluate the impact of initiatives to promote school implementation of environmental changes. While direct observation represents a gold standard data collection method, for assessment of school environmental characteristics such procedures are expensive and impractical at a population level [18,19].

Population based assessments of school environment characteristics in Australia [20], the United States of America [21] and Canada [22] have mostly relied on Principal or School Administrators completion of telephone or paper surveys. Despite the use of such measures, few have been validated [18]. For example, the validity of the nutritional and physical activity environmental characteristics of schools in Australia, as reported in large, recurrent surveys of Australian school Principals [20] has not been reported. Similarly, the United States National School Health Policies and Programs Study (SHPPS) of school health policies and practices has reported the reliability but not the validity of the questionnaires measuring school practices [23]. An unpublished validation study of the Canadian School Health Environment Survey (SHES) [24] is one of the few studies that have reported the validity of school survey tools. The report found high percent agreement (range 70%-100%) between direct observation and combined school administrator/teacher “best response” for questions related to existing physical activity facilities and healthy eating programs and promotions. Lower agreement was found for availability of healthy vending machines and promotion of active transport (50.0% and 66.7% percent agreement respectively). The 2007 study was limited by it being conducted in 14 schools, of which eight catered for primary school aged children, and each school being observed for one day [18]. More recently, two school physical activity environment audit tools developed in the United Kingdom have been validated by direct observation. In both cases the tools focussed only on the physical activity or sport environment of schools [19,25]

Given the limited evidence regarding the validity of self reported school characteristics regarding the promotion of student healthy eating and physical activity, we undertook a study to determine the validity of Principal reported nutrition and physical activity characteristics of primary schools.

## Methods

Approval to conduct the study was obtained from Hunter New England Area Health Service (HNEAHS) Human Research Ethics Committee (no. 06/07/26/4.04) and The University of Newcastle (H-2008-0343).

### Design and setting

A descriptive study, comparing cross sectional data from two measures of the healthy eating and physical activity environment of primary (children 5–12 years of age) and central schools (children aged 5-18 years of age) was undertaken. The study was conducted in the context of a large scale child obesity prevention initiative in the Hunter New England region of New South Wales (NSW), Australia [26].

### Participants

#### Principal self-report

All primary schools in the study region were approached to participate in a 20-minute telephone survey of school environment characteristics related to the promotion of child healthy eating and physical activity.

#### Observational data

A sub-sample of schools participating in the telephone survey were approached to participate in the collection of observational data regarding school environment characteristics.

### Data collection procedures

#### Principal self-report

A database of all primary and central schools across the state was generated from information provided by relevant school bodies [27-29]. All schools were eligible to participate in the study other than special purpose schools catering for students with special needs, juvenile justice or schools serving children who are hospitalized. Principals were sent a letter inviting them to participate in the study. Two weeks after receipt of the letter, Principals were telephoned by a trained research assistant who confirmed school eligibility, sought consent to participate in the study and scheduled a time for a telephone interview. At the completion of the telephone survey Principal consent was sought for observational data to be collected. The survey was conducted in late 2010.

To identify a comprehensive list of school environment factors for inclusion in the telephone survey the research team conducted a review of the literature regarding school environmental factors associated with student diet, physical activity, sedentary behaviour or excessive weight gain, as well as items from similar tools. A draft survey tool was circulated to an expert advisory group (n = 10) of health promotion practitioners, school education directors, local Principals and teachers, academics, parents and dietitians. The items were amended based on advisory group member’s review and feedback regarding the scope of the survey, the appropriateness of survey items and comprehension and then pilot tested with five primary school Principals to check acceptability and comprehension. The final survey tool (*School Environment Assessment Tool (SEAT))* consisted of 65 survey items. A copy of the survey tool is available as supplementary material.

#### Observational data

Pre-service teachers on placement in primary schools in the region as part of their fourth year University teacher training program were recruited to collect the observational data. Pre-service teachers were informed of the school practices to be observed at the beginning of their 10-week placement. To ensure they had an adequate amount of time to observe all practices they were surveyed in week 9 of their 10 week placement, away from school premises to ensure that there was no influence from school personnel, using a paper-based form. The pre-service teachers were surveyed two weeks after the completion of the telephone survey by the school Principal. They were not aware of the Principal’s response to the telephone survey. If the pre service teachers did not observe any of the practices, for example fundraising activities, they were instructed to provide a ‘don’t know’ response. The paper-based observational data collection form was developed using equivalent items from the telephone survey. The form was piloted with 15 pre-service teachers to establish its utility and acceptability, with minor amendments subsequently being made.

### Measures

#### Food availability

The SEAT required Principals to report (yes/ no/ don’t know/ refused) if any of the following foods or drinks were sold at the school from the (a) canteen, (b) vending machines or (c) via fundraisers: fruit; vegetables; water; regular soft drinks (not diet); diet soft drinks; fruit juice; other sweetened drinks including cordials, energy drinks, flavoured mineral waters etc.; confectionary; sweet and savoury biscuits/ cakes/ muffins/ donuts or muesli bars; potato crisps or other salty snacks such as twisties or corn chips; deep fried foods; ice creams covered in chocolate; other ice creams, iceblocks or frozen desserts.

#### Physical activity opportunities

The SEAT required Principals to report on the physical activity facilities and equipment and organised physical activities that students had available to them whilst at the school. These included;

(a) Physical activity facilities and equipment- Principals were asked to report whether the school had any of the following that students can access everyday (yes/ no/ don’t know) and if yes when can they access them (recess only/ lunch only/ both recess and lunch);

– Large asphalt areas suitable for games e.g. basketball/ netball;

– Large playing fields suitable for games e.g. soccer, football;

– Indoor activity spaces e.g. multi-purpose rooms and gymnasiums;

– Playground markings e.g. hopscotch, wall targets etc.;

– Fixed playground equipment such as climbing structures, swings etc.;

– Sports equipment provided by the school e.g. basketballs, netballs, skipping ropes etc.

(b) Organised physical activities- Principals were asked if, during recess or lunch, the school had organised physical activities e.g. dance/ gymnastics (yes/ no/ don’t know); whether they are co-ordinated by the school or an outside agency; and if teachers participate or join in.

(c) Small screen recreation- Principals were asked to report whether students were able to access small screen recreation (yes/ no/ don’t know) during recess or lunch; before or after school; during wet weather sport.

(d) School sport- Principals were asked to report if both infants and primary students had sport.

#### School characteristics

During the telephone interview, Principals were asked to report the number of students attending the school. School type (Government or non-Government (Catholic or Independent) and the postcode of the locality of the school were obtained from school websites.

### Analysis

Statistical analyses were performed using SAS v9.2 (SAS Institute Inc., Cary, NC, USA). Descriptive statistics were used to describe school characteristics and the proportion of Principals and pre-service teachers who reported “yes” to any of the school practices. The reported number of enrolled students in each school was used to categorise school size as: ‘small (1–159 students); ’medium’ (160–450 students); or ‘large’ (451+ students) based on the Department of Education and Communities School Directory [30]. Schools with postcodes ranked socio-economically in the top 50% of New South Wales [31] were categorised as schools of ‘higher socio-economic status’ while those in the lower 50% were categorised as schools of ‘lower socio-economic status’. School postcode areas were also used to categorise the school’s locality as either ‘rural’ (those schools in outer regional, remote and very remote areas), or ‘urban’ (those in regional cities and inner regional areas) [32].

Two measures of agreement were calculated to describe the validity of Principal report. First, percent agreement between Principal report and observation was calculated for each item. As recommended, agreement levels ≥80% were considered ‘strong’ [33].

Whilst percent agreement is widely used and easily interpreted [34], it does not correct for agreement over and above what would be expected by chance [35]. As such, the second and primary measure of validity was assessed using the Kappa statistic [35]. Prevalence Adjusted and Bias Adjusted Kappa (PABAK) was calculated and reported where the prevalence of a particular school environment characteristics was found to be ≥ 75% (or less than 25%). Non adjusted Kappa statistics were calculated and reported for items with prevalence between 26% and 74%. Benchmarks suggested by Landis and Koch [36] were used to classify agreement: < 0.00 = poor, 0.00 – 0.20 = slight, 0.21 – 0.40 = fair, 0.41 – 0.60 = moderate, 0.61 – 0.80 = substantial and 0.81 – 1.0 = almost perfect.

## Results

### Sample

In total, 384 of 403 Principals participated in the CATI. Of these, 63 schools had a pre-service teacher on placement in the Hunter New England Region of NSW and hence were eligible to participate. Of these, seven school Principals did not consent to having their pre-service teacher participate in data collection leaving a maximum potential sample of 56 (Principal consent rate = 89%). There was no statistically significant difference in terms of school size, socio-economic status or rurality for schools who consented to having their pre-service teachers complete the survey and those that did not. On the day of data collection with the pre-service teachers, three refused to participate and 11 were absent (pre-service teacher response rate = 75%). This provided 42 matched pairs of CATI-observational data.

Of the telephone survey respondents, 35 were Principals who had been in their position on average 5.9 years (SD 4.0 years), with the remaining participants being Deputy or Assistant Principals (all respondents hereafter are referred to as Principals). The majority of the schools were medium sized (57%) (equal proportions of small and large schools participated (21%)), Government schools (67%), located in urban (98%) and in higher socio-economic areas (67%).

### Validity of Principal self report

The validity of principal responses to the telephone survey items are presented in Table 1. As both Principals and observers reported that no vending machines were located in the schools, percent agreement and kappa scores were 100% and are not presented for these 26 items.

**Table 1:** Validity of survey items assessing the nutrition and physical activity environment of primary schools

**Aspect of the school environment**	**% Yes (Principal)**	**% Yes (Pre-service teacher)**	**% Agree**	**Kappa/PABAK (95% CI)**
***Food sold in canteen***				
• Fruit	95	83	79	0.57 (0.26, 0.79)^a^
• Vegetables	90	62	57	−0.06 (−0.26, 0.14)
• Water	93	86	88	0.76 (0.49, 0.92)^a^
• Regular soft drinks	0	24	76	0.52 (0.21, 0.76)^a^
• Diet soft drinks	2	24	74	0.48 (0.16, 0.72)^a^
• Fruit juice	98	93	90	0.81 (0.55, 0.95)^a^
• Other sweetened drinks	48	55	69	0.38 (0.11, 0.66)
• Confectionary	19	43	62	0.16 (−0.1, 0.43)
• Sweet and savoury biscuits	55	64	52	0.02 (−0.28, 0.32)
• Potato crisps	57	69	55	0.04 (−0.25, 0.34)
• Deep fried foods	0	31	69	0 (0, 0)
• Ice creams covered in chocolate	0	21	79	0.57 (0.26, 0.79)^a^
• Other ice creams	95	88	88	0.76 (0.49, 0.92)^a^
***Food sold in fundraising***				
• Fruit	19	5	81	0.62 (0.32, 0.83)^a^
• Vegetables	24	7	79	0.57 (0.26, 0.79)^a^
• Water	12	0	88	0.76 (0.49, 0.92)^a^
• Regular soft drinks	17	7	81	0.62 (0.32, 0.83)^a^
• Diet soft drinks	14	2	88	0.76 (0.49, 0.92)^a^
• Fruit juice	17	0	83	0.67 (0.37, 0.86)^a^
• Other sweetened drinks	14	7	88	0.76 (0.49, 0.92)^a^
• Confectionary	31	12	67	0.06 (−0.21, 0.33)
• Sweet and savoury biscuits	29	10	67	−0.02 (−0.26, 0.22)
• Potato crisps	12	7	90	0.81 (0.55, 0.95)^a^
• Deep fried foods	2	7	95	0.90 (0.68, 0.99)^a^
• Ice creams covered in chocolate	0	7	93	0.86 (0.61, 0.97)^a^
• Other ice creams	10	5	90	0.81 (0.55, 0.95)^a^
***Physical activity facilities accessible at recess and lunch***				
• Large asphalt areas	95	90	86	0.71 (0.43, 0.89)^a^
• Large playing fields	83	88	81	0.62 (0.32, 0.83)^a^
• Indoor activity spaces	19	24	67	0.01 (−0.29, 0.32)
• Playground markings	95	90	90	0.81 (0.55, 0.95)^a^
• Fixed playground equipment	67	83	74	0.48 (0.16, 0.72)^a^
• School sports equipment	60	93	57	−0.02 (−0.2, 0.15)
***Organised physical activities at recess and lunch***				
• Organised physical activity	10	10	100	1 (1, 1)^a^
• Do teachers join in	40	40	100	1 (1, 1)^a^
***Students access to small screen recreation***				
• During Recess/Lunch	7	64	37	0 (−0.12, 0.12)
• Before school	33	19	59	0.02 (−0.28, 0.31)
• During wet weather sport	5	31	73	0.46 (0.14, 0.72)^a^
***School sport***				
• K-2 have sport	88	85	83	0.67 (0.37, 0.86)^a^
• 3-6 have sport	95	90	90	0.81 (0.55, 0.95)^a^

^a^Adjusted Kappa used if prevalence greater than or equal to 75% (or less than 25%); *CI* confidence interval.

#### Percent agreement

Per cent agreements for the remaining 39 items, ranged from 37% to 100%. Twenty items (51%) were found to have percent agreements greater than or equal to 80%. The questions related to student’s access to small screen recreation had substantially lower per cent agreement estimates.

#### Kappa statistics

PABAK were calculated for 27 of the 39 items as they had prevalence ≥ 75% (or less than 25%). Based on such analyses, three items had poor agreement (< 0.00), eight had slight agreement (0.00-0.20), one had fair agreement (0.21-0.40), seven had moderate agreement (0.41-0.60), 11 had substantial agreement (0.61-0.80) and nine had almost perfect agreement (0.81-1.0).

With respect to the food environment, foods sold through fundraisers had the highest Kappa/PABAK estimates with 10 items having substantial or better agreement. Kappa/ PABAK scores for foods sold in the canteen varied (range −0.6-0.81), although most had slight to moderate agreement.

Across the four aspects of the physical activity environment that were measured, Kappa/PABAK scores were, in general, quite good. Three items within “physical activity facilities” were found to have moderate or better agreement and two items within “organised physical activities” were found to have perfect agreement (school has supervised or instructed physical activity at recess and lunch and whether teacher’s participate or join in the organised activities at recess and lunch).

## Discussion

To the author’s knowledge this is the first study to assess the validity of a Computer Assisted Telephone Interview (CATI) survey of principal reported primary school nutrition and physical activity environment characteristics using direct observation as the criterion standard. The findings of the study suggest that Principals can accurately report on a large number of food and physical activity environmental characteristics of their school.

The study found that more than half of the survey items assessed had moderate, substantial or almost perfect agreement. Substantial or greater agreement was found for most items that assessed foods sold through fundraising, physical activity facilities of the school, and organised physical activities that occurred at recess and lunch. Such findings are consistent with the findings of the few other validation studies of the healthy eating and or physical activity and sports environment of schools [24,25]. The high level agreement found within these domains may be for a number of reasons. The physical activity facilities are permanent fixtures in the school and the organised activities occur regularly and are visible activities it is likely Principals can more accurately report on these. Similarly, as Principal approval is needed for school fundraising activities to occur, and the fact they may occur only once or twice a term, it is likely Principals are able to easily recall these events and the foods sold. However, these findings should be viewed with some caution, as no time period was specified, it is possible Principals may have been reporting on fundraising events throughout the year whilst pre-service teachers could only report on fundraising events which occurred during the nine weeks they were in the school.

In contrast, foods sold through the canteen and student’s access to small screen recreation were found to have lower agreement. This is similar to findings of the Canadian validation study which found low agreement for school administrators and teachers being able to report on the schools provision of healthy vending machines [24]. As school canteens sell a wide variety of foods, and as products sold through canteens are frequently modified according to student demand and product availability and season, recalling foods sold through canteens may represent a significant challenge for Principals. Collection of school policies and parent communication strategies (e.g. newsletter items) could be undertaken in future studies to help validate such items that are difficult to quantify by direct observation but are known to be associated with student’s physical activity and healthy eating such as school policies and parent engagement strategies [14].

A strength of this study was the use of observational data over a nine week period. However, this methodology could be enhanced by tasking pre service teachers with observations prospectively rather than retrospectively. Given the differences in school systems, the generalizabilty of the SEAT to schools outside of Australia may be limited and thus may need to be adapted for international use and have its validity tested in these jurisdictions. Furthermore, given the challenges of collecting observational data the study utilised a convenience sample of pre-service teachers as observers. Nonetheless, randomisation of pre-service teachers to schools would have strengthened the design.

## Conclusion

Given the significant influence schools have on children’s behaviours this tool may provide researchers, policy makers and practitioners with an efficient method of assessing the nutrition and physical activity environment of primary schools and evidence as to whether obesity prevention interventions are achieving expected outcomes and the need for on-going intervention.

## Abbreviations

SEAT: School Environment Assessment Tool; CATI: Computer Assisted Telephone Interview.

## Competing interests

The authors declare that they have no competing interests.

## Authors’ contributions

NN, LW, PJM, CB and JW conceptualized the study design and measures. Data were collected by NN. DB performed the statistical analysis; NN, DB, LW interpreted data. NN led the development of the manuscript and all authors contributed to drafts and read and approved the final manuscript.

## References

[B1] Story M Nanney MS Schwartz MB Schools and obesity prevention: creating school environments and policies to promote healthy eating and physical activity Milbank Q 2009 87 1 71 100 10.1111/j.1468-0009.2009.00548.x 19298416 PMC2879179

[B2] Swinburn B Egger G Raza F Dissecting obesogenic environments: the development and application of a framework for identifying and prioritizing environmental interventions for obesity Prev Med 1999 29 563 70 10.1006/pmed.1999.0585 10600438

[B3] Fein AJ Perceived environment and physical activity in youth Int J Behav Med 2004 11 3 135 142 10.1207/s15327558ijbm1103_2 15496341

[B4] Kubik MY The association of the school food environment with dietary behaviors of young adolescents Am J Public Health 2003 93 7 1168 73 10.2105/AJPH.93.7.1168 12835204 PMC1447928

[B5] French SA Food environment in secondary schools: À La Carte, vending machines, and food policies and practices Am J Public Health 2003 93 7 1161 1167 10.2105/AJPH.93.7.1161 12835203 PMC1447927

[B6] Waters E Interventions for preventing obesity in children Cochrane Database Syst Rev 2011 12 CD001871 pub3 10.1002/14651858.CD001871 22161367

[B7] Jaime PC Lock K Do school based food and nutrition policies improve diet and reduce obesity? Prev Med 2009 48 1 45 53 10.1016/j.ypmed.2008.10.018 19026676

[B8] Neumark-Sztainer D School lunch and snacking patterns among high school students: associations with school food environment and policies Int J Behav Nutr Phys Act 2005 2 14 1 7 16209716 10.1186/1479-5868-2-14 PMC1266392

[B9] Sallis JF The association of school environments with youth physical activity Am J Pub Health 2001 91 618 620 11291375 10.2105/ajph.91.4.618 PMC1446652

[B10] Ridgers ND Long-term effects of a playground markings and physical structures on children’s recess physical activity levels Prev Med 2007 44 5 393 397 10.1016/j.ypmed.2007.01.009 17335891

[B11] Stratton G Mullan E The effect of multicolor playground markings on children’s physical activity level during recess Prev Med 2005 41 5–6 828 833 16137756 10.1016/j.ypmed.2005.07.009

[B12] Verstraete SJ Increasing children’s physical activity levels during recess periods in elementary schools: the effects of providing game equipment Eur J Public Health 2006 16 4 415 9 10.1093/eurpub/ckl008 16431866

[B13] Wechsler H Using the school environment to promote physical activity and healthy eating Prev Med 2000 31 S121 S137 10.1006/pmed.2000.0649

[B14] Centers for Disease Control and Prevention School Health Guidelines to Promote Healthy Eating and Physical Activity 2011 Atlanta: Division of Population Health School Health Branch 1 76

[B15] de Silva-Sanigorski A Government food service policies and guidelines do not create healthy school canteens Aust N Z J Public Health 2011 35 2 117 121 10.1111/j.1753-6405.2010.00694.x 21463405

[B16] New South Wales Auditor-General’s Report Performance Audit Physical Activity in Government Primary Schools Department of Education and Communities 2012 Sydney, NSW: Audit Office of NSW, Editor

[B17] NSW Department of Health and NSW Department of Education and Training Fresh Tastes @ School NSW Healthy Canteen Strategy Canteen Menu Planning Guide 2006 Sydney NSW: NSW Department of Health

[B18] McGraw SA Measuring implementation of school programs and policies to promote healthy eating and physical activity among youth Prev Med 2000 31 S86 S97 10.1006/pmed.2000.0648

[B19] Jones NR School environments and physical activity: the development and testing of an audit tool Health Place 2010 16 776 783 10.1016/j.healthplace.2010.04.002 20435506 PMC3820999

[B20] Hardy LL NSW Schools Physical Activity and Nutrition Survey (SPANS) 2010: Full Report 2010 Sydney: NSW Ministry of Health, Editor

[B21] Kyle TM Methods: school health policies and programs study 2006 J Sch Health 2007 77 8 398 407 10.1111/j.1746-1561.2007.00227.x 17908100

[B22] Allison KR Adlaf EM Structured opportunities for student physical activity in ontario elementary and secondary schools Can J Publ Health 2000 91 5 371 375 10.1007/BF03404810 PMC6979646 11089292

[B23] Brener ND Kann L Smith TK Reliability and validity of the school health policies and programs study 2000 questionnaires J Sch Health 2003 73 1 29 37 10.1111/j.1746-1561.2003.tb06556.x 12621721

[B24] Manske S Centre for Behavioural Research and Program Evaluation Pilot phase of the 2007–2008 school health environment survey: technical report october 24, 2008 2008 Waterloo, Ontario: University of Waterloo

[B25] Fairclough SJ The physical education and school sport environment inventory preliminary validation and reliability Environ Behav 2012 44 1 50 67 10.1177/0013916510388495

[B26] NSW Department of Health Good for Kids.Good for Life 2009 http://www.goodforkids.nsw.gov.au/Parents

[B27] NSW Department of Education and Training NSW School Locator 2008 http://www.schools.nsw.edu.au/schoolfind/locator

[B28] Catholic Education Commission NSW Catholic Schools Directory 2008 http://stage.cecnsw.catholic.edu.au/2009/lisa.asp

[B29] Private Schools Directory Private Schools Directory NSW 2008 http://www.privateschoolsdirectory.com.au/search.php

[B30] New South Wales Department Of Education and Training 2009 DET Directory 2009 Sydney, NSW: Department of Education and Training 1 204

[B31] Australian Bureau of Statistics (ABS) Technical Paper:Census of Population and Housing: Socio-Economic Indexes For Australia (SEIFA). Cat. no 2039.0.55.001 2001 Canberra: Commonwealth of Australia

[B32] Australian Bureau of Statistics (ABS) Statistical Geography Volume 1- Australian Standard Geogrphical Classification (ASGC). Cat. no 1216.0 2006 Canberra: Commonwealth of Australia

[B33] Hartmann DP Considerations in choice of interobserver reliability estimates J Appl Behav Anal 1977 10 1 103116 10.1901/jaba.1977.10-103 PMC1311156 16795538

[B34] Henderson KE Validity of a measure to assess the child-care nutrition and physical activity environment J Am Diet Assoc 2011 111 9 1306 1313 10.1016/j.jada.2011.06.011 21872693 PMC4172575

[B35] Byrt T Bishop J Carlin JB Bias, prevalence and kappa J Clin Epidemiol 1993 46 5 423 429 10.1016/0895-4356(93)90018-V 8501467

[B36] Landis JR Koch GG The measurement of observer agreement for categorical data Biometrics 1977 33 1 159 174 10.2307/2529310 843571

